# Preparation of Polyurethane Solid–Solid Phase-Change Material with Soft Segment for Application on Temperature-Regulating Fabrics

**DOI:** 10.3390/ma19204296

**Published:** 2026-10-12

**Authors:** Xiran Qiao, Shaofeng Lu, Qiancheng Yang, Songcong Lin, Mingqiang Wang, Wenzhao Shi, Chao-Qun Ma

**Affiliations:** 1 School of Textile Science and Engineering, Xi’an Polytechnic University, No. 19 Jinhuanan Road, Xi’an 710048, China; qiao@xpu.edu.cn (X.Q.);; 2 Key Laboratory of Advanced Dyeing and Finishing Technology of Shaanxi Higher Education Institutes, Xi’an Polytechnic University, Xi’an 710048, China; 3 Fujian Techwork Textile Co., Ltd., Quanzhou 362700, China

**Keywords:** composite soft segment, solid–solid phase-change material, water-borne polyurethane, temperature-regulating fabric, cotton fabric

## Abstract

The emergence of water-borne polyurethane solid–solid phase-change materials (PCMs) has overcome the problems of core material leakage in solid–liquid phase-change materials. However, water-borne polyurethane solid–solid phase-change materials also have issues such as poor heat storage performance and limited temperature regulation range. This study used polycaprolactone diol (PCL-diol) and poly(ethylene glycol) (PEG) as the composite soft segments, and selected dicyclohexylmethane-4,4′-diisocyanate (HMDI) and glycerol (GL) as the hard segments, to prepare a PEG-PCL-HMDI PCM. By adjusting the ratio of the composite soft and hard segments, the content of the soft segment, and the molar ratio of the monomers, the phase-change temperature was precisely controlled and the heat storage performance was synergistically improved. After the PEG-PCL-HMDI PCM had finished being applied onto the surface of cotton fabric, the temperature-regulating fabric exhibited a significant temperature-buffering effect in the 25–35 °C human activity temperature range, with the heating rate reduced and the cooling rate delayed. This study provides a new material system and process scheme for the development of intelligent textiles with both efficient energy storage and precise temperature regulation functions.

## 1. Introduction

In the context of the escalating global energy crisis and environmental issues, the development of efficient and energy-saving materials has become a research hotspot in the field of materials science [1]. Solid–solid phase-change materials (SSPCMs) do not undergo a liquid phase transition during the phase-change process and possess significant advantages such as small volume change, no leakage risk, and strong thermal stability. They are becoming ideal candidate materials for energy storage and temperature regulation [2,3]. In the textile industry, SSPCMs can endow textiles with intelligent temperature regulation functions, maintaining a comfortable body temperature by absorbing or releasing latent heat [4]. They show broad application prospects in outdoor clothing, medical textiles, home textiles, etc. [5], which is of great significance for promoting the transformation of the textile industry towards a green and intelligent direction [6].

However, the currently commercialized solid–solid phase-change materials still have some drawbacks that restrict their large-scale promotion in the textile industry [7]. First, the phase-change temperatures of most traditional SSPCMs are above 40 °C, which do not match the comfortable temperature range of the human body (25–32 °C). This temperature range cannot effectively meet the temperature regulation requirements of daily textiles [8]. Second, the phase-change enthalpy of some materials is low, and the energy storage density is insufficient. Therefore, the temperature regulation effect of SSPCMs is not long-lasting and is difficult to adapt to complex and variable environmental temperature changes [9]. Finally, SSPCMs have poor compatibility with textile fibers. During spinning or post-treatment processes, SSPCMs are prone to agglomeration and detachment, further limiting their practical application [10].

In terms of controlling the phase transition temperature, through molecular structure design, such as introducing flexible segments and changing the types and positions of substituents, precise reduction in the phase transition temperature can be achieved [11]. In terms of enhancing the phase transition heat capacity, methods like copolymerization and grafting are employed to construct multicomponent copolymer systems, increasing the regularity of molecular chains, thereby improving the energy storage capacity of the material [12]. In terms of improving compatibility, surface modification techniques are utilized, such as coating nanoparticles and grafting active fiber groups, to enhance the bonding force between the solid–solid phase transition materials and the fiber interface [13,14]. Although these studies have achieved certain progress, there are still problems such as complex modification processes, high costs, and limited performance improvement [15].

In response to this technical bottleneck, Jinming Chang et al. [16] successfully expanded the working temperature range of an SSPCM to 27.4–73.2 °C by using blending strategies for polyethylene glycols (PEGs) with different molecular weights, such as PEG-200, PEG-400, PEG-600, and PEG-1000. This system demonstrates unique advantages in the field of wide-temperature-range thermal management, but its latent heat storage density of 16.5 J/g is far below the commercial application requirements (>50 J/g). To increase the latent heat storage density, Lu et al. [17] modified the material with trimethylolpropane (TMP) and methacrylic acid hydroxyethyl ester (HEMA), and then cross-linked it with water-borne polyurethane (WPU) with PTMG as the soft segment, generating a multi-cross-linked, two-component phase-change water-borne polyurethane (PCWPU). The highest heat enthalpy of the PCWPU film can reach 90.3 J/g. However, in practical use, SSPCMs inevitably undergo cracking or fracture, and the phase-change materials usually lack healing properties and cannot be recycled for further processing. Therefore, developing an SSPCM with a low phase-change temperature, high latent heat storage density, and self-healing properties remains a key scientific issue that urgently needs to be addressed in the field of functional materials for textile thermal regulation [18].

**Figure 1:**
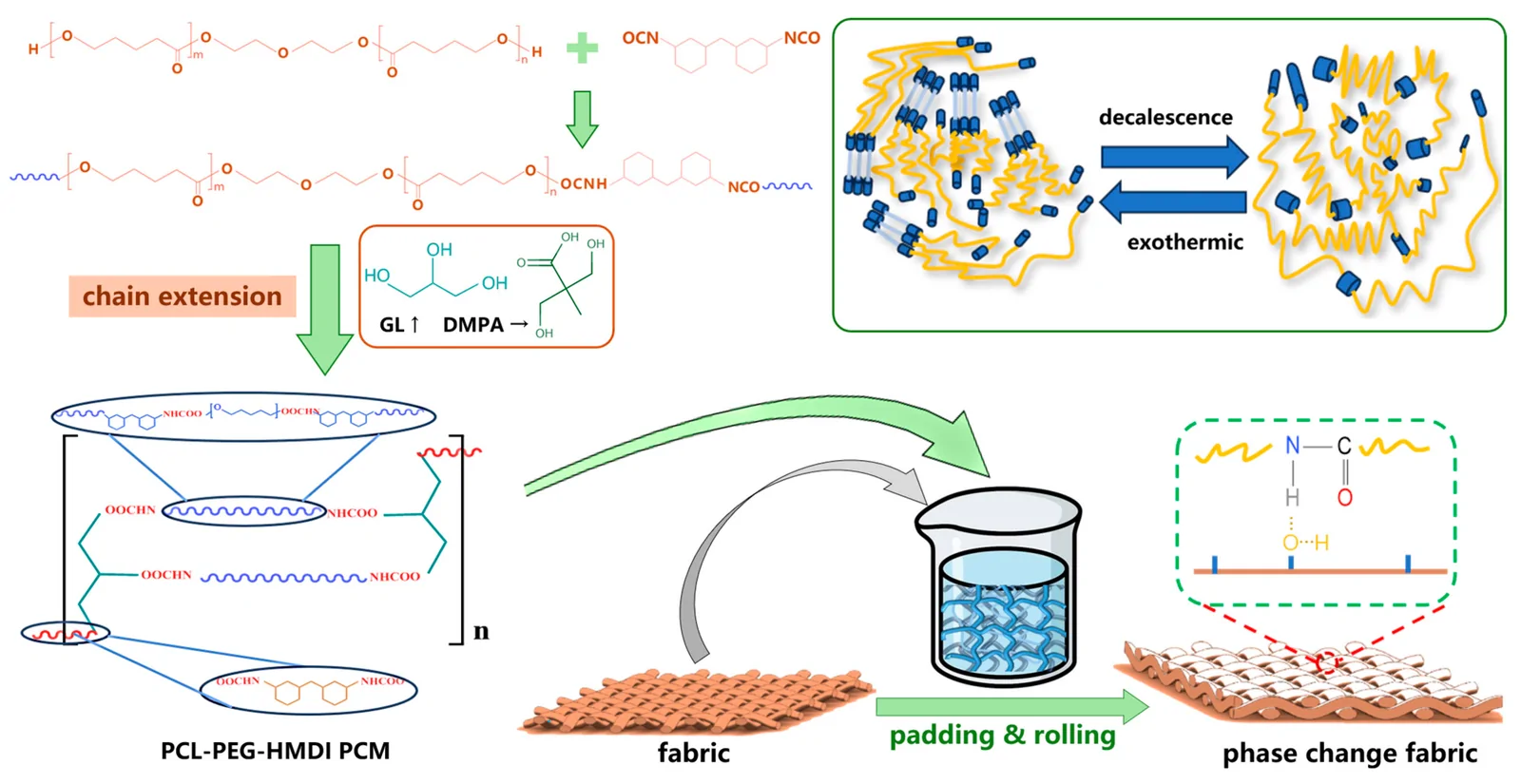
Process of preparing PEG-PCL-HMDI PCM and temperature-regulating fabric.

Based on this, in this study, a PEG-PCL-HMDI PCM was prepared as shown in Figure 1. Polycaprolactone diol (PCL-diol) and poly(ethylene glycol) were used as the composite soft segments, while dicyclohexylmethane-4,4′-diisocyanate (HMDI) and glycerol (GL) were used as the hard segments. HMDI has a highly symmetrical molecular structure, which makes the molecular chains more regular in arrangement and provides stronger crystallization ability. During the phase transition process, it can store and release more heat, improving the energy storage capacity of the material. GL itself contains three hydroxyl groups (-OH), which can react with isocyanate HMDI through multi-functional reactions to form a three-dimensional-network cross-linked structure, significantly increasing the cross-linking density of polyurethane, thereby significantly reducing the phase transition temperature of the phase-change material [19].

By adjusting the ratio of the composite soft and hard segments, the content of the soft segment, and the molar ratio of the monomers, precise control of the phase transition temperature and a synergistic improvement in the heat storage performance were successfully achieved. In this study, phase-change materials were combined with cotton fabrics to create functional fabrics with temperature regulation capabilities. Novel shape-stable water-borne polyurethane phase-change materials were fabricated using PEG and PCL as composite soft segments. Variation in the PEG/PCL composition enables controllable regulation of phase-change temperature, and the as-prepared PCMs can be directly utilized for cotton fabric modification. This research result not only provides technical support for the application of solid–solid phase-change materials in the textile field, but also offers new ideas and methods for the development of intelligent textiles.

## 2. Experiment

### 2.1. Materials

Poly(ethylene glycol) (PEG, M_n_ = 2000) was purchased from Damao Chemical Reagent Factory in Tianjin, China. Polycaprolactone diol (PCL-diol, M_n_ = 500) was obtained from Jining Hongming Chemical Reagent Co., Ltd. (Jining, China). Dicyclohexylmethane-4,4′-diisocyanate (HMDI) was bought from Xuzhou Yihuiyang New Materials Co., Ltd. (Xuzhou, China). Ethylenediamine (EDA), 2,2-Bis(hydroxymethyl)propionic acid (DMPA) and triethylamine (TEA) were purchased from Tianjin Tianli Chemical Reagent Co., Ltd. (Tianjin, China). Glycerol (GL) and *N*,*N*-Dimethylformamide (DMF) were bought from Tianjin Fuyu Fine Chemical Co., Ltd. (Tianjin, China).

HMDI, DMF, and GL were added in advance to microporous silica for dehydration treatment for more than 3 days; PEG and PCL-diol were filtered under vacuum at a temperature above 120 °C for more than 2 h to remove small molecules such as water.

### 2.2. Preparation of Phase-Change Material

Different proportions of PEG and PCL were placed into a three-necked flask and dried under vacuum at 110 °C for 2 h. HDMI was added, and the reaction temperature was maintained at 80 °C, the speed of the electronic stirrer was set at 180 r/min, and the reaction was carried out for 2 h. DMPA was then added and reacted for another 2 h to obtain the polyurethane prepolymer. The temperature was subsequently lowered to 45 °C, and TEA was added for neutralization, with the reaction continued for 30 min. Finally, the amount of deionized water was calculated according to a solid content of 30%, and emulsification was performed for 30 min. In addition, EDA was added to consume the remaining –NCO groups. The reaction system was protected by nitrogen throughout the whole process. Appropriate amounts of DMF were added to reduce the viscosity of the system during the reaction.

The number of moles of HDMI added is twice the total of PEG and PCL-diol. Details of the quality of the individual units are presented in Table 1. If there are any additional samples with different monomer ratios, they will be indicated in the data results.

GL and DMPA were added and reacted for 2 h to obtain the polyurethane prepolymer. The temperature was then lowered to 45 °C, and TEA was added for a 30 min neutralization reaction. Finally, 30% deionized water was added and emulsified for 30 min. Additionally, EDA was added to consume the remaining -NCO. The entire reaction was carried out under nitrogen protection. Appropriate amounts of DMF were added to reduce the viscosity of the entire reaction system. The resulting PCM was labeled as PEG-PCL-HMDI PCM.

The phase-change material without PEG and HMDI was used as a control group, marked as PCL. The phase-change material without HMDI was used as another control group, marked as PEG-PCL.

### 2.3. Preparation of Temperature-Regulating Fabric

When the PCM is attached to the cotton fabric, the molar ratio of PEG:PCL-diol is 19:1. The molar ratio of PEG-PCL:HMDI:GL is 3:6:2. The prepared PCM was dispersed at different concentrations. Then, the cotton fabric was immersed in the solutions for 30 min. The fabric was then subjected to two-dip two-nip padding for finishing, and then dried at 110 °C for 30 min.

### 2.4. Characterization

The morphology of the PCM samples was observed at different temperatures using a KYKY-2800B scanning electron microscope produced by Beijing Zhongke Keyi Co., Ltd. (Beijing, China). The surface of the samples was gold-coated, and the test voltage was 25 kV.

The crystallization processes of different PCM samples were observed and recorded. The samples were placed on a glass slide that had been cleaned with alcohol, and then the glass slide was placed on a DM2700P thermal stage POM produced by Leica Camera AG (Wetzlar, Germany). The temperature was raised to 70 °C, and the temperature was maintained for 20 min. Then, the temperature was lowered to room temperature. The crystalline morphology of the PCM phase transition process was observed under constant temperature conditions. The prepared materials were molded into regular samples, and then placed in an oven at a temperature higher than the phase transition temperature for 30 min. The macroscopic morphological changes in the samples were observed and photographed.

The chemical structure of the prepared samples was tested and characterized using an FTIR analyzer (Perkin Elmer, MA, USA), with the wavenumber range being 4000–500 cm^−1^. The crystallization performance of the PCM samples was tested using a SmartLabSE X-ray diffractometer produced by Japan Rigaku Corporation (Tokyo, Japan). The scanning range was 10–50°, with the copper target as the test material and the test speed set at 5°/min.

The thermal stability of the prepared samples was tested using the TGA2 thermogravimetric analyzer produced by Mettler-Toledo International Inc. (Columbus, OH, USA). The weight of the sample was 5 to 10 mg. The temperature range was 20–600 °C, with a heating rate of 10 °C/min and nitrogen protection. The samples were characterized using a DSC1 differential scanning calorimeter (Mettler Toledo, Shanghai, China). The test temperature range was −20–80 °C, with a heating and cooling rate of 5 °C/min, and nitrogen protection was employed. The same experimental conditions were maintained during each heating–cooling cycle.

An RX700 infrared thermal imaging instrument, produced by Dongguan Fenvan Electronics Co., Ltd. (Dongguan, China), was used to monitor and take photos of the surface temperature of the PCM membrane. Then, the temperature around the PCM membrane was raised from 0 °C to 65 °C, kept at this temperature for 10 min, and then lowered back to 0 °C. This process was carried out to simulate the changes in the PCM membrane in a working environment. The temperature of the PCM membrane was monitored and recorded using a digital thermometer produced by Hengshui Chuangji YIqiyibiao Co., Ltd. (Hengshui, China).

The infrared thermal imaging instrument was used to monitor and take photos of the surface temperatures of the blank fabric and the temperature-regulating fabric. Then, the temperatures around the blank fabric and the temperature-regulating fabric were raised from −10 °C to 50 °C, kept at this temperature for 10 min, and then lowered back to −10 °C. This process was carried out to simulate the changes in the temperature-regulating fabric in a working environment. The surface temperatures of the fabrics were monitored and recorded using a digital thermometer.

## 3. Results and Discussion

### 3.1. Structure and Morphology

**Figure 2:**
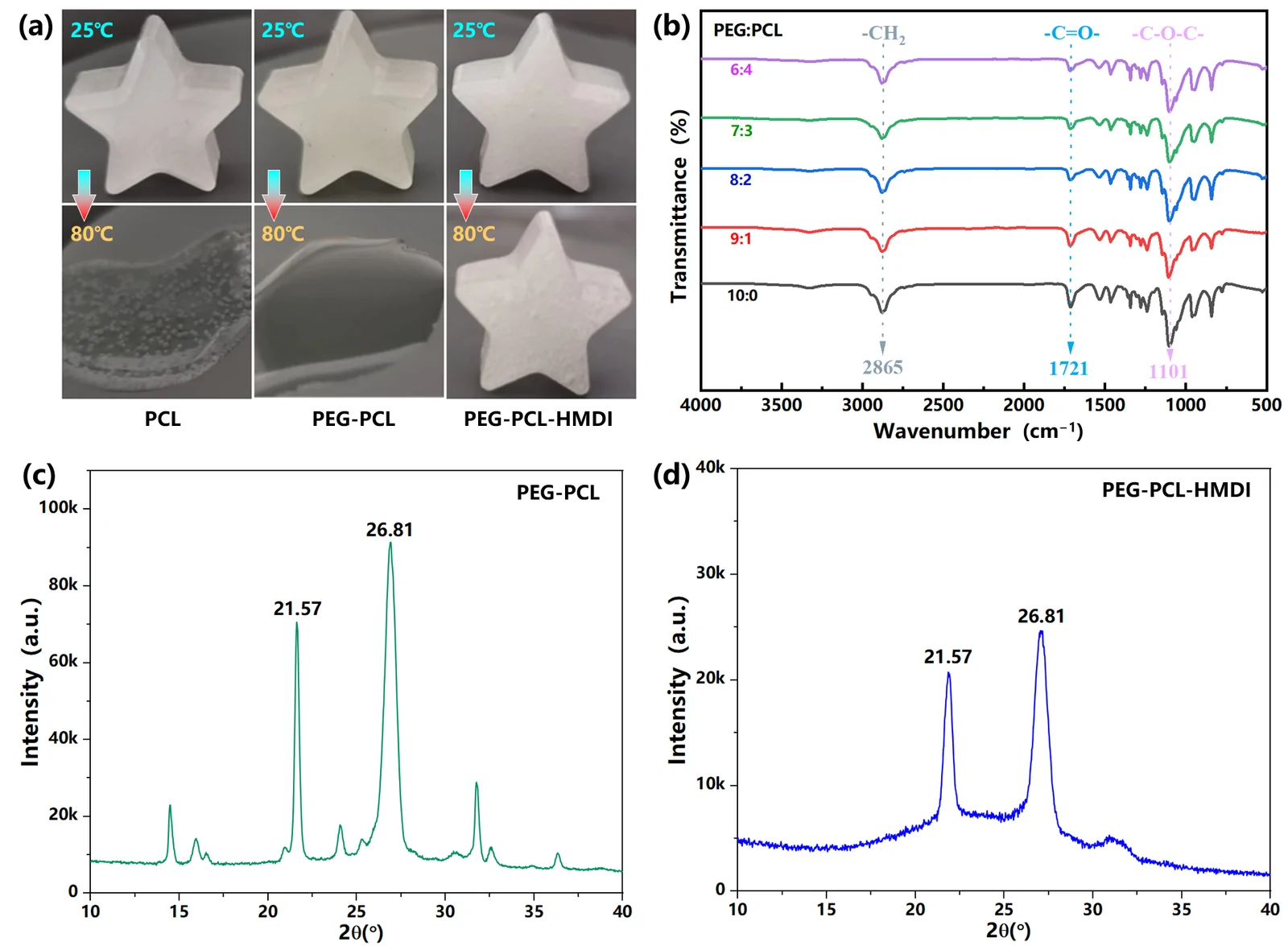
(**a**) Morphological changes in PEG, PEG-PCL and PEG-PCL-HMDI PCM before and after phase transition, (**b**) FTIR spectrum of PEG-PCL-HMDI PCM with different PCL/PEG ratios, and XRD patterns of (**c**) PEG-PCL and (**d**) PEG-PCL-HMDI PCM.

The leakage test is the most direct method to determine whether a phase-change material has a stable phase-change shape. Different samples, including PCL, PEG-PCL and the PEG-PCL-HMDI PCM, were placed in environments of 25 °C (room temperature) and 80 °C for about 30 min. The prepared materials were molded into regular samples using a star-shaped mold, and then placed in an oven at a temperature higher than the phase transition temperature of the samples. The samples were kept at this temperature for 30 min, then removed, and the macroscopic morphological changes in the samples were observed and photographed. The morphology of the materials changed significantly, as shown in Figure 2a.

Different materials exhibited different morphological changes at different temperatures, reflecting their distinct phase-change characteristics. PEG and PEG-PCL composite materials were in a solid state at 25 °C and had already melted significantly when placed in an environment of 80 °C. This might be due to the weak forces of the PEG molecular chains. The PEG-PCL-HMDI PCM was in a solid state at 25 °C and could maintain a solid state when placed in an environment of 80 °C, demonstrating the excellent solid–solid phase-change characteristics and shape stability of the prepared PEG-PCL-HMDI PCM. Even at higher temperatures, it can maintain a solid state well, avoiding the problem of liquid leakage during the phase-change process. This is because PCL-diol and PEG form a molecular chain entanglement or interpenetrating network structure when HMDI exists in the molecule. This structure can effectively limit the deformation of the material during the phase-change process, thereby maintaining the phase-change stability of the phase-change material.

Additionally, the PEG-PCL composite material is condensed between GL and HMDI through intermolecular forces, serving as a cross-linking point, like a series of “nodes” connecting the soft segments, enabling the PEG-PCL-HMDI PCM to maintain a solid shape as a whole. Even if the soft segments undergo a crystalline-to-amorphous transformation, due to the cross-linking effect of the hard segments, the material will not exhibit a macroscopic liquid flow phenomenon like a solid–liquid phase change, but will always remain in a solid state, demonstrating the characteristics of solid–solid phase change [20].

Figure 2b presents the FTIR analysis spectrum of the PEG-PCL composite material. In the FTIR spectrum, the absorption peaks at 1102 cm^−1^ and 1058 cm^−1^ can be attributed to the stretching vibration of the -C-O-C- bond [21]. For HMDI, the characteristic infrared absorption peak of -NCO- exists at 2243 cm^−1^. The absorption peak at 1714 cm^−1^ is the characteristic absorption peak of the carbonyl group (C=O) [22,23]. The absorption peak at 1529 cm^−1^ originates from the coupling absorption peak of the C-N bond stretching vibration and the N-H bond deformation vibration.

Figure 2c,d show the XRD patterns of the PEG-PCL composite material and PEG-PCL-HMDI PCM. The patterns exhibit two distinct and similar characteristic diffraction peaks at 21.57° and 26.81°, indicating that both the PEG-PCL blend and PEG-PCL-HMDI PCM exist in a crystalline state at room temperature [24]. Additionally, it was found that the intensity of the characteristic peaks of the PEG-PCL-HMDI PCM is significantly lower than that of PEG-PCL, which is due to the introduction of the hard segment, which restricts the composite soft segment and causes the crystalline particles of the composite soft segment to become smaller and the crystallinity to decrease.

The solid–solid phase transition of the material can be observed through a polarizing microscope during the transition between crystalline and amorphous states. The study of crystal morphology provides insight into the phase transition ability of the phase transition material. Under the polarizing microscope, the growth process of spherical crystals of the PEG-PCL composite material and PEG-PCL-HMDI PCM can be observed.

**Figure 3:**
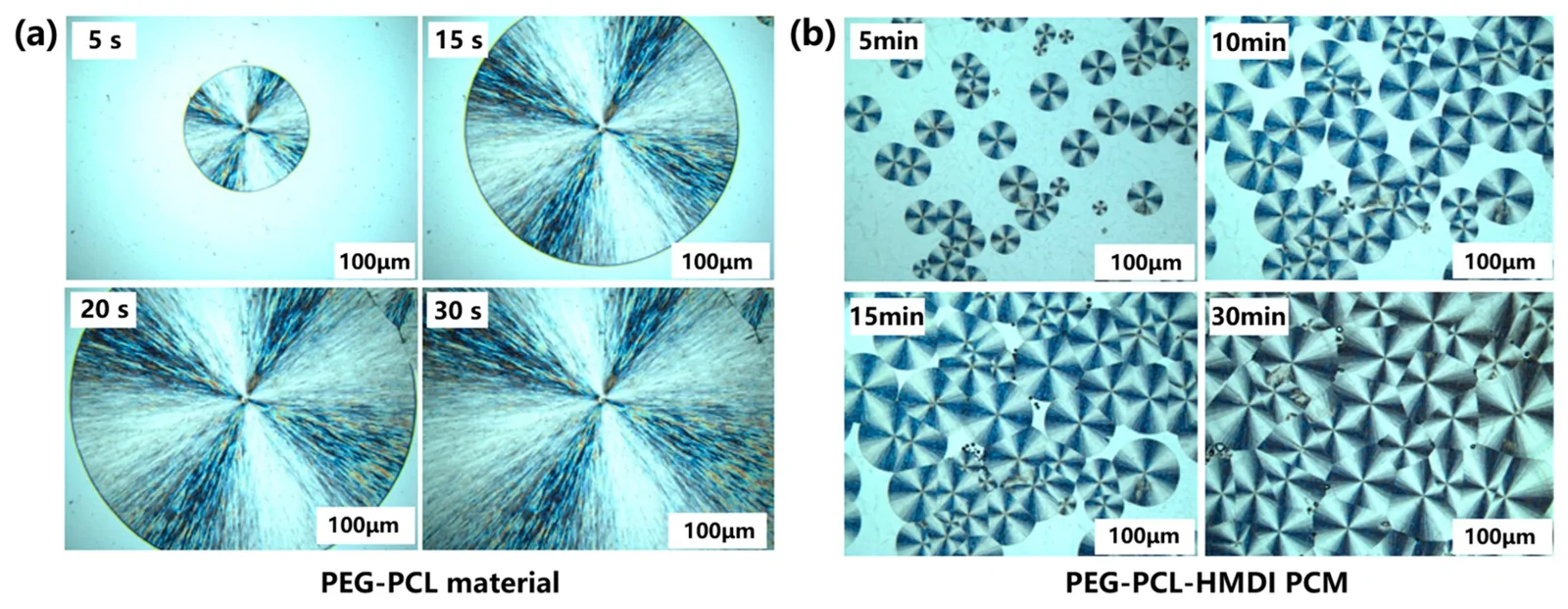
(**a**) Crystal growth process during isothermal crystallization of PEG-PCL composite material. (**b**) Crystal growth process during isothermal crystallization of PEG-PCL-HMDI PCM.

Figure 3 shows the microscopic photos of PEG-PCL and the PEG-PCL-HMDI PCM under constant temperature conditions. In the POM images of both at room temperature, obvious black cross-extinction patterns [25] were observed, indicating that molecules of various molecular weights of PEG-PCL and the PEG-PCL-HMDI PCM prepared therefrom exist in spherical crystal form at room temperature, and also suggesting that the crystalline form of PEG was not changed during the preparation of the PEG-PCL-HMDI PCM [26].

In the early stage of crystallization, the PEG/PCL crystals initially exhibited spherulitic crystallization. The growth process of the spherical crystal went through a process of nucleation first and then growth. The growth rate of each spherical crystal in PEG-PCL was basically the same until it collided with the adjacent spherical crystal.

Compared with the PEG-PCL composite material, Figure 3b shows that the spherical crystal size in the PEG-PCL-HMDI PCM is relatively smaller, and its growth rate is significantly lower than that of PEG-PCL. This is because the cross-linking of the polymer has led to the crystallization of the soft segment, resulting in a lower crystallinity of the PEG-PCL-HMDI PCM compared to the PEG-PCL [27]. This is also the reason why the phase transition enthalpy of the PEG-PCL-HMDI PCM is lower than that of the PEG-PCL composite material. However, since the spherical crystal size of the PEG-PCL-HMDI PCM is smaller than that of PEG-PCL, it is more conducive to the growth of the spherical crystal in space, thereby forming a more complete crystal structure.

### 3.2. Phase-Change Heat Energy Analysis

**Figure 4:**
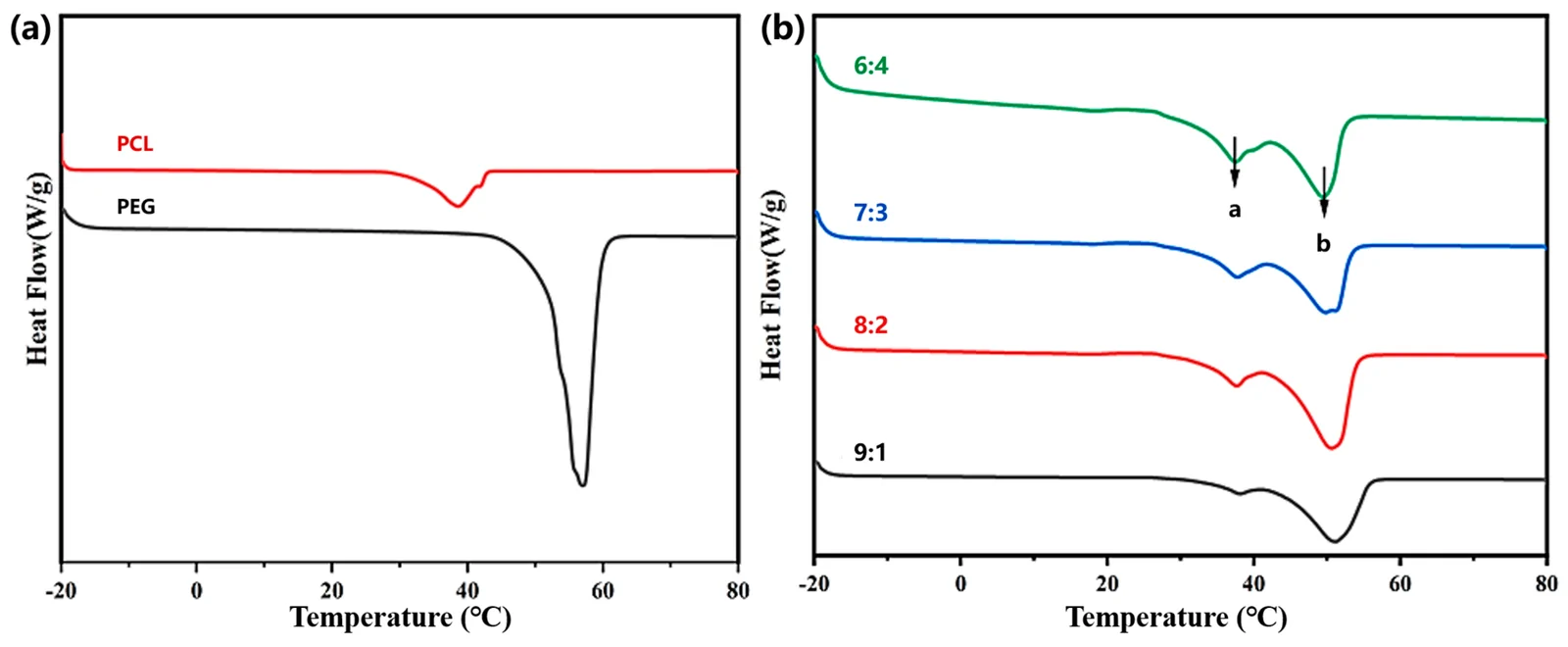
The influence of PEG/PCL ratio on the heat storage performance of the composite. (**a**) Heat storage performance of PCL-diol and PEG. (**b**) Heat storage performance under different PEG/PCL ratios.

The PEG and PCL-diol were uniformly mixed in mass ratios of 9:1, 8:2, 7:3, and 6:4 using an ultrasonic oscillator to obtain the complexes. Then, the complexes were subjected to DSC testing, and the results are shown in Figure 4. The results also demonstrate the influence of the PEG/PCL ratio on the heat storage performance of the complexes in Table 2.

In Figure 4b, the melting peak of pure PCL-diol is labeled as peak a, and the melting peak of pure PEG is labeled as peak b. According to Figure 4b and Table 2, due to the different polarities of the two substances, peak a of PCL-diol and peak b of PEG appear simultaneously in the complex. Moreover, the melting temperature of the b peak in the complex is significantly lower than that of the pure PEG peak, which is because PCL-diol has a lower melting temperature, causing PCL-diol and PEG to fuse with each other, resulting in the formation of a new melting peak of PEG. The melting temperature of this peak decreases significantly compared to that before the complex formation. As the ratio of PEG/PCL-diol decreases from 9:1 to 6:4, the enthalpy value at peak a gradually increases, the enthalpy value at peak b gradually decreases, and the total enthalpy value also gradually decreases. The melting temperatures of both peaks show no significant decrease.

**Figure 5:**
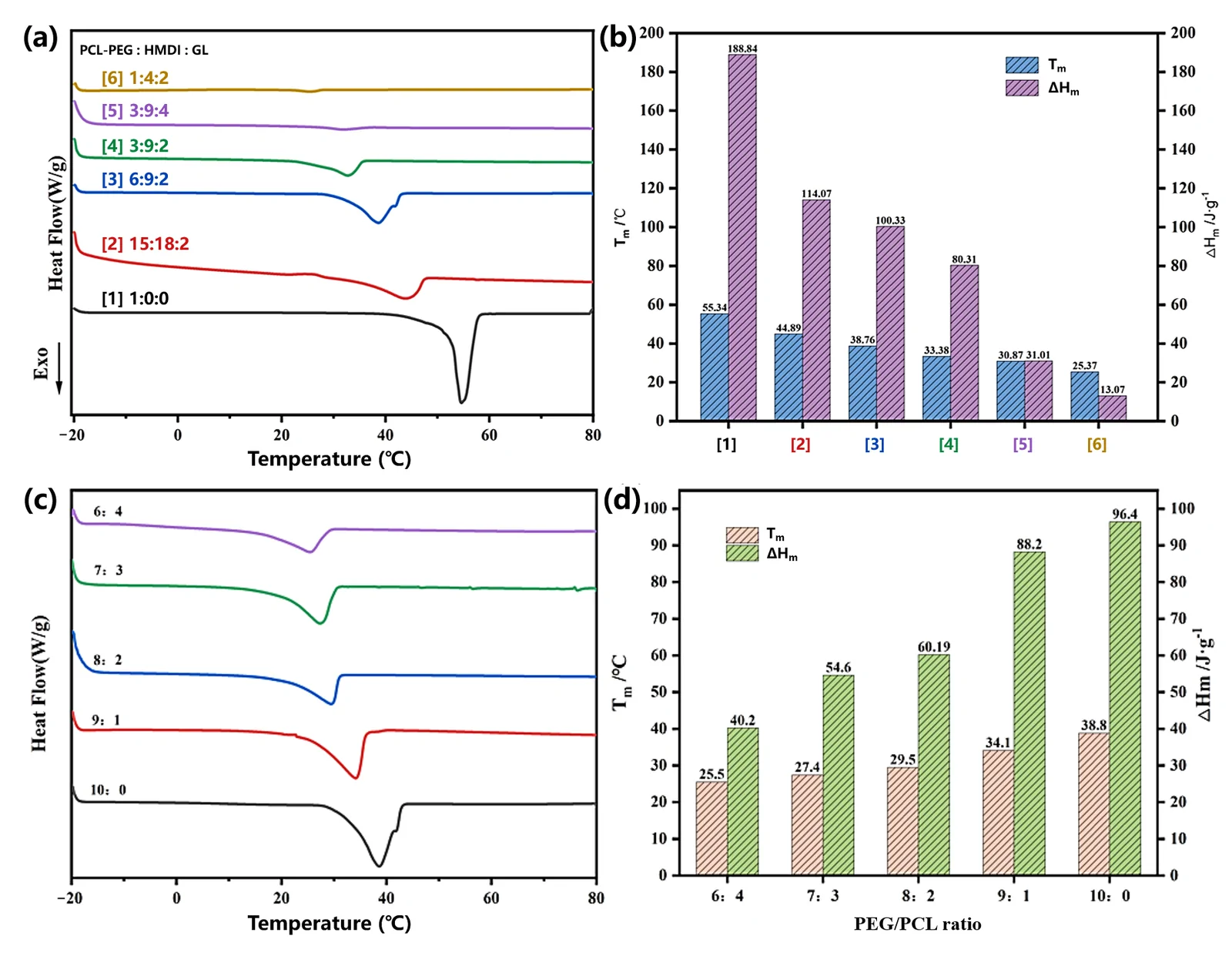
(**a**) DSC curves of PEG-PCL-HMDI PCM, (**b**) heat storage performance diagram of PEG-PCL-HMDI PCM, (**c**) DSC curves of PEG-PCL-HMDI PCM, and (**d**) phase transition temperature and phase transition enthalpy of PEG-PCL-HMDI PCM.

The DSC curve in Figure 5a shows that the difference in the heat flow intensity of the curve reflects the different enthalpy of the phase transition. The larger the area of the endothermic peak, the higher the corresponding phase transition enthalpy, indicating that the material has different heat storage capabilities. Different molar ratios of the formulation significantly affect the heat storage performance of the material.

With the incorporation of the hard segment, the melting temperature and heat enthalpy of the PEG-PCL-HMDI PCM show a downward trend. When the content of the soft segment is between 61.85% and 82.71%, although a PEG-PCL-HMDI PCM with a melting temperature ranging from 25.37 °C to 38.76 °C can be prepared, the heat enthalpy is only 13.07 J/g to 100.33 J/g. However, when the content of the soft segment is between 82.71% and 85.95%, the melting temperature is 38.76 °C to 44.89 °C, and the heat enthalpy is 100.33 J/g to 114.07 J/g.

This phenomenon is primarily attributed to PEG, the soft segment, whose highly flexible chains and high crystallinity govern the phase-change heat-storage behavior of the material. HMDI (hard segment) reacts with GL (chain extender) to form a rigid network that restricts PEG chain motion. As the hard-segment fraction increases, this confinement strengthens, reducing PEG crystallinity and consequently lowering both the phase-change temperature and enthalpy. Mechanistically, hard-segment amide groups form hydrogen-bonded rigid structures that enhance intermolecular forces, disrupting the intrinsic ordered crystalline arrangement of PEG and enabling melting at a lower temperature; the concomitant decrease in crystallinity further diminishes heat-storage capacity. The single melting peaks observed in the thermograms are assignable to PEG segments, and their low-temperature shift and reduced enthalpy reflect the decreased relative content of PEG-based soft segments.

The molecular network constructed by HMDI and GL restricts the phase transition range of the soft segments. The higher the proportion of the hard segments, the denser the network structure, and the more significant the constraint of the cross-linking points on the soft segments, resulting in a lower phase transition temperature [28].

The DSC curve shows that the difference in the heat flow intensity of the curve reflects the different enthalpy of phase transition. The larger the area of the endothermic peak, the higher the corresponding enthalpy of phase transition, indicating that the material has different abilities to store heat. After preparing the PEG-PCL-HMDI PCM by combining PEG and PCL-diol, the phase transition temperature and enthalpy significantly improve.

Figure 5c,d shows that as the PCL-diol proportion increases, both the phase transition temperature and enthalpy show a downward trend with increasing PCL-diol content. This indicates that the increase in PCL-diol content weakens the heat storage capacity of the phase transition material, and the effect of the composite ratio on the regulation of heat enthalpy is significant. PEG and PCL-diol form molecular chain entanglements, and at the same time, the rigid network constructed by the hard segment will restrict the movement of the soft segment. With increasing PCL-diol content, the disorder of the soft-segment molecular chains increases, and the binding effect of the hard segment on the soft segment becomes more significant, further hindering the crystallization of PEG, reducing the energy required for the phase transition process, and ultimately resulting in a decrease in the phase transition temperature and enthalpy with increasing PCL-diol content. In addition, the molecular chain structure of PCL-diol is different from that of PEG, and its introduction will disrupt the ordered crystallization of PEG. With increasing PCL-diol content, the crystalline area of PEG decreases, the crystallinity reduces, and it also leads to a decrease in the phase transition temperature and enthalpy.

**Figure 6:**
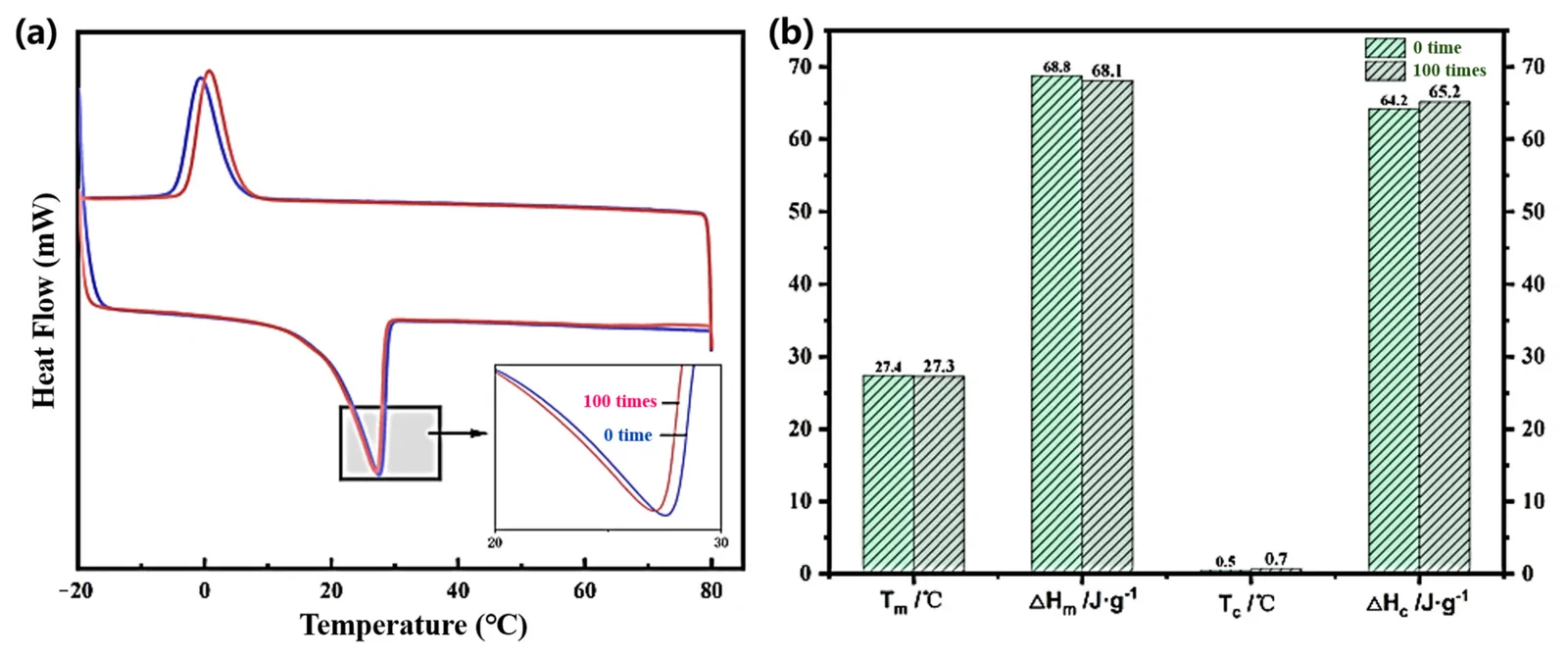
(**a**) DSC curves of the phase-change material before and after 100 heating–cooling cycles, and (**b**) performance change diagram of the phase-change material before and after cycling.

Figure 6 shows the DSC curves of the PEG-PCL-HMDI PCM phase-change material before and after 100 heating–cooling cycles. The PEG-PCL-HMDI PCM was heated from −20 °C to 80 °C and then cooled back to −20 °C. Before and after 100 heating–cooling cycles, the positions of the endothermic peak (T_m_) and the exothermic peak (T_c_) were basically the same, and the difference in peak areas was also small, with no significant deviation. This indicates that after multiple heating–cooling cycles, the phase transition temperature of the material remained stable and was not significantly affected by the internal structure changes due to the cycles, thus not affecting the phase-change behavior. Due to the entanglement of PEG and PCL-diol molecular chains, a cross-linked network structure is formed. In addition, the rigid network structure composed of the hard segments (HMDI, GL) within the phase-change material also plays a restraining role during heating–cooling cycles. Even after multiple temperature changes, the cross-linking points can still maintain the relative stability of the molecular chains, preventing the degradation or structural disorder of the soft segments (PEG/PCL-diol) due to repeated thermal motion, thereby ensuring the stability of the phase transition temperature and heat capacity [29,30].

### 3.3. Temperature-Regulating Fabric

**Figure 7:**
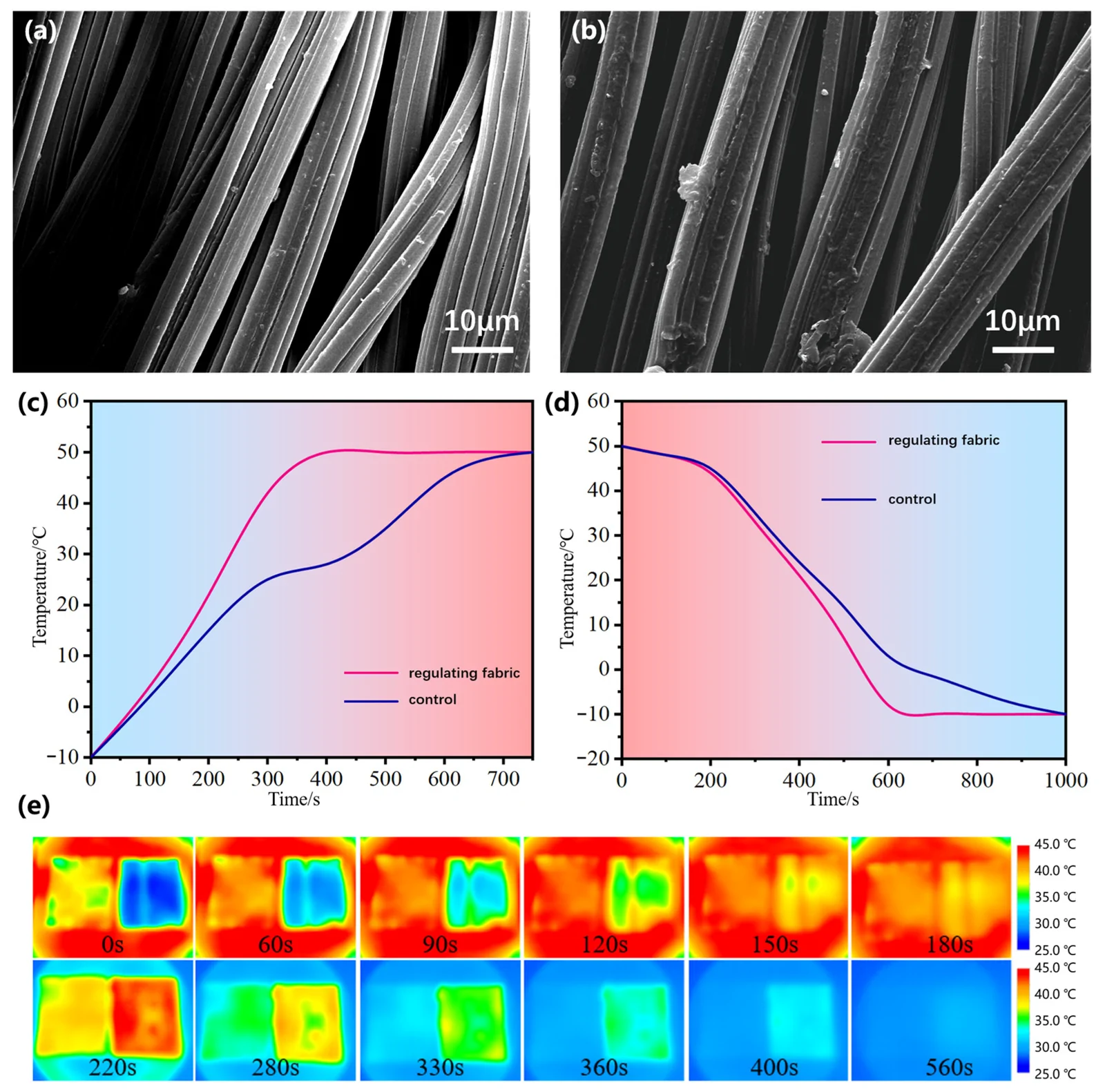
(**a**) SEM morphology images of the blank fabric and (**b**) the temperature-regulating fabric. The time–temperature curves of the blank fabric and the temperature-regulating fabric during (**c**) heating and (**d**) cooling. (**e**) The infrared thermal images of the blank fabric and the temperature-regulating fabric, with the left side being the blank control and the right side being the temperature-regulating fabric.

Figure 7 shows the morphology and properties of the temperature-regulating fabric. SEM images of the untreated and treated fabrics are shown in Figure 7a; the surface of the blank fabric’s fibers is smooth and free of impurities, and the fibers are arranged naturally, presenting the original structural characteristics of the fabric. In Figure 7b, obvious granular or sheet-like attachments appear on the surface of the temperature-regulating fabric fibers, breaking the original smooth form of the fibers. This indicates that the water-borne polyurethane solid–solid phase-change material (PEG-PCL-HMDI PCM) has successfully been loaded onto the fabric fibers. It endows the fabric with temperature-buffering capability. At the same time, the fiber structure has not been severely damaged, indicating that the finishing process, while ensuring the material loading, retains the basic performance of the fabric, laying a structural foundation for its practical application.

To simulate the changing process of the fabric in a working environment, the temperature around the blank fabric and the temperature-regulating fabric was synchronized from −10 °C to 50 °C, and then maintained at 50 °C for 10 min before being lowered back to −10 °C. During this period, the surface temperature of the fabric was monitored and recorded in real time using a digital thermometer. As shown in Figure 7c,d, when the temperature of the treated fabric reached approximately 27.45 °C, the temperature rise was significantly delayed and at a slower rate, indicating that the water-borne polyurethane solid–solid phase-change material (PEG-PCL-HMDI PCM) loaded in the fabric underwent a phase change during the heating process.

During this process, the temperature-regulating fabric absorbs and stores heat, effectively delaying the increase in fabric temperature and demonstrating the heat-absorbing and temperature-regulating function of the phase-change material. This is mainly due to the transformation of the crystalline state in the PEG and PCL-diol macromolecular chains to an amorphous state. As time progresses, when all the crystalline states eventually turn into amorphous states, the temperature-regulating fabric is unable to continue absorbing energy, and thus, the surface temperature of the temperature-regulating fabric will become the same as that of the blank fabric. When the temperature drops, the temperature-regulating fabric releases heat to slow down the rate of temperature decrease on the fabric surface. This demonstrates that the prepared temperature-regulating fabric has excellent heat storage and temperature-regulating performance [31,32]. The heat absorption and release enables the temperature to remain relatively constant for a certain period of time. It provides a good temperature “buffer zone” between human skin and the fabric.

The infrared thermal imager recorded the real-time temperature of the sample during this process. Figure 7e illustrates the heating and cooling processes of the blank fabric and the temperature-adjusted fabric in the infrared thermal imaging. Figure 7e indicates that after the fabrics make contact with the heating plate, there is a certain temperature difference between the blank fabric and the temperature-regulating fabric, which may be due to the different responses of the two materials to the ambient temperature.

During the heating stage (0–184 s), the temperatures of both the blank fabric and the temperature-regulating fabric were increasing. However, the temperature increase rate of the temperature-regulating fabric was relatively slower, and the color change was not as obvious as that of the blank fabric. This indicates that the water-borne polyurethane solid–solid phase-change material, when applied to the fabric, can absorb environmental heat for phase change. The water-borne polyurethane solid–solid phase-change material delays the increase in fabric temperature and plays a role in temperature regulation.

During the cooling stage (220–560 s), the temperature of the blank fabric dropped rapidly and its color turned blue quickly. The temperature of the temperature-regulating fabric decreased relatively slowly, indicating that the phase-change material releases stored heat during the cooling process. The water-borne polyurethane solid–solid phase-change material slows down the decrease in fabric temperature and keeps the fabric temperature relatively stable.

The temperature-regulating fabric was loaded with the PEG-PCL-HMDI PCM. It exhibited a buffering effect on temperature changes during both the heating and cooling processes. This finding confirms that the PCM can effectively regulate fabric temperature and maintain thermal stability in fluctuating environments. By contrast, the blank fabric lacked this function. This behavior stems from the ability of the PCM to store and release heat during the solid–solid phase transition. This heat storage and release mechanism provides key performance support for temperature-regulating fabrics in temperature control applications, such as functional clothing and smart textiles.

## 4. Conclusions

In this study, we successfully prepared a water-borne polyurethane solid–solid phase-change material with both efficient energy storage and temperature-buffering capability by constructing a PEG/PCL-diol composite soft-segment and cross-linking network structure.

The experimental results show that the introduction of PCL-diol significantly reduced the phase transition temperature of the PEG-based material, and by adjusting the PEG/PCL-diol mass ratio, the phase transition temperature and heat enthalpy were synergistically optimized. The cross-linking network structure, by restricting the movement of the soft-segment molecular chains, enabled the material to remain in a solid state at 60 °C and showed a phase transition temperature drift of <0.5 °C and a heat enthalpy attenuation of <5% after 100 heating–cooling cycles, demonstrating excellent cycle stability.

After the material was applied to cotton fabric, the temperature-regulating fabric exhibited a significant temperature-buffering effect in the human activity temperature range of 25~35 °C, with the heating rate reduced and the cooling rate delayed. The mechanism study indicates that the hard segment forms physical cross-linking with the soft segment through hydrogen bonds, inhibiting excessive crystallization of PEG, and the semi-crystalline property of PCL-diol further optimizes the uniformity of the material’s thermal response.

This study provides a new material system for the development of intelligent textiles suitable for human temperature regulation, and its design concept can be extended to the preparation of other functional phase-change materials.

## Figures and Tables

**Table 1:** Synthesis of PEG-PCL-HMDI PCM using different proportions of raw materials.

Sample Number	Mass Ratio (PEG:PCL-Diol)	PEG/g	PCL-Diol/g	HMDI/g	GL/g
1	9:1	27	3	10.23	1.11
2	8:2	24	6	12.59	1.48
3	7:3	21	9	14.95	1.75
4	6:4	18	12	17.31	2.03

**Table 2:** The influence of PEG/PCL-diol ratio on the heat storage performance of the composite material.

PEG/PCL-Diol Ratio	*T*_a_/°C	*T*_b_/°C	Δ*H*_a_ ^1^/J·g^−1^	Δ*H*_b_ ^2^/J·g^−1^	Δ*H*_a_ + Δ*H*_b_/J·g^−1^
9:1	38.7	50.3	8.2	168.5	176.7
8:2	38.6	50.3	13.4	150.1	163.5
7:3	38.5	50.2	24.2	131.3	155.5
6:4	38.4	50.2	32.6	108.9	141.5

^1^ ΔH_a_ represents the enthalpy value at peak a in Figure 4b. ^2^ ΔH_b_ represents the enthalpy value at peak b in Figure 4b.

## Data Availability

The original contributions presented in this study are included in the article. Further inquiries can be directed to the corresponding author.
